# Interleukin 24 Promotes Mitochondrial Dysfunction, Glucose Regulation, and Apoptosis by Inactivating Glycogen Synthase Kinase 3 Beta in Human Prostate Cancer Cells

**DOI:** 10.3390/cells14050357

**Published:** 2025-02-28

**Authors:** Anastassiya Kim, Sual Lopez, Simira Smith, Alphons Sony, Jennifer Abreu, Columba de la Parra, Moira Sauane

**Affiliations:** 1 Department of Biological Sciences, Herbert H. Lehman College, City University of New York, 250 Bedford Park Boulevard West, New York, NY 10468, USA; anastassiya.kim@lehman.cuny.edu (A.K.); sual.lopez@lc.cuny.edu (S.L.); simira.smith@lc.cuny.edu (S.S.); alphons.sony@lc.cuny.edu (A.S.); jennifer.abreu2@lc.cuny.edu (J.A.); 2 The Graduate Center, City University of New York, 365 Fifth Avenue, New York, NY 10016, USA; columba.delaparra@lehman.cuny.edu; 3 Department of Chemistry, Herbert H. Lehman College, City University of New York, 250 Bedford Park Boulevard West, New York, NY 10468, USA

**Keywords:** glycogen synthase kinase-3 beta, protein kinase A, Interleukin 24, mitochondria dysfunction, p38 MAPK, glucose, metabolism, apoptosis, prostate cancer

## Abstract

Interleukin 24 (IL-24) is a tumor-suppressing protein currently in clinical trials. We previously demonstrated that IL-24 leads to apoptosis in cancer cells through protein kinase A (PKA) activation in human breast cancer cells. To better understand the mechanism by which IL-24 induces apoptosis, we analyzed the role of glycogen synthase kinase-3 beta (GSK3β), a highly conserved serine/threonine kinase in cancer cells and a downstream target of PKA. Our studies show for the first time that GSK3β is inhibited following IL-24 treatment in human prostate cancer cells. We showed that the inhibition of GSK3β is mediated through PKA activation triggered by IL-24. IL-24 decreases the phosphorylation of glycogen synthase, substantially activating glycogen synthase and decreasing intracellular glucose levels. Notably, the expression of a constitutively active form of GSK3β abolishes the effect of IL-24. These results demonstrate a previously unrecognized role of IL-24 in apoptosis mediated through GSK3β regulation and its possible implications for metabolic stress, mitochondria dysfunction, and apoptosis. Future studies should precisely delineate the most effective combinations of IL-24 as a GSK3β inhibitor with cytotoxic agents for prostate and other cancers. GSK3β inhibition disrupts average glucose utilization in cancer cells, potentially creating metabolic stress that could be exploited therapeutically.

## 1. Introduction

Interleukin 24 (IL-24) is an immunomodulatory molecule belonging to the IL-10 cytokine family that signals through the two heterodimeric receptors IL22R/IL-20R2 and IL-20R1/IL-20R2 [1,2,3]. It is a cytokine produced by immune and non-immune cells and exerts various functions [4,5,6]. Lymphocytes, monocytes, melanocytes, epithelial stem cells, and dermal-keratinocytes produce IL-24 [4,5,6]. IL-24 plays essential roles in multiple conditions: it exerts autoimmune effects in psoriasis [7,8], atopic dermatitis [9], and chronic spontaneous urticaria [10]; it inhibits the proliferation and migration of keratinocytes during wound repair, and it coordinates dermal repair and re-epithelialization [5].

Several pre-clinical and clinical studies have indicated that IL-24 exhibits tumor suppression activities [2,11,12,13]. These tumor suppression activities include angiogenesis inhibition [3,14], inhibition of metastasis and invasion [15,16], and triggering apoptosis in cancer-specific cells [3,17,18,19,20,21,22]. Studies on the role of IL-24 in cancer have shown that this cytokine can exert its tumor suppression effect through various molecular mechanisms [2]. We and others have previously demonstrated that IL-24 leads to apoptosis in cancer cells by endoplasmic reticulum (ER) stress activation [17,23,24,25], reactive oxygen species (ROS) accumulation [25,26], p38 MAPK activation [17,25,27], mitochondrial dysfunction [18,25,28], and ceramide production, while not affecting normal cells [23,29]. Additionally, we have shown that IL-24-activated protein kinase A (PKA) induces apoptosis in breast cancer cells [17]. IL-24 increases intracellular cAMP (cyclic adenosine monophosphate) levels, activating PKA and phosphorylating its substrates [17].

The PKA inactivates multiple substrates, including p90 ribosomal S6 kinase, p70 ribosomal S6 kinase, protein kinase B pathways, and Glycogen synthase kinase—3 alpha (GSK3α) and beta (GSK3β) [17]. This study focuses on GSK3β due to its emerging potential as a therapeutic target in prostate cancer. Notably, the immunohistochemical analysis of 499 prostate cancer cases revealed that elevated cytoplasmic (but not nuclear) GSK3β expression correlates with aggressive disease features, such as advanced stage, lymph node metastasis, extracapsular extension, higher Gleason scores, and reduced recurrence-free survival over 12 years [30]. Additionally, the high cytoplasmic expression of GSK3β is associated with high-risk prostate cancer cases [31].

Glycogen synthase kinase-3 (GSK3) plays a role in cancer progression and in cancers resistant to radio-, chemo-, and targeted therapies [32,33,34]. GSK3 consists of GSK3α and GSK3β, both ubiquitously expressed and highly conserved [35]. GSK3β is a well-known inhibitor of β-catenin in the Wnt signaling pathway, which regulates gene expression associated with cellular differentiation, proliferation, and survival. Notably, research suggests that the selective inhibition of GSK3β has antitumor effects independent of Wnt signaling [31,32,33,34,35,36,37]. Furthermore, inhibiting GSK3β has been shown to enhance the effectiveness of cancer therapies while safeguarding normal tissues from treatment-induced damage [34,35,36]. Despite its therapeutic importance, Lithium remains the only FDA-approved GSK3β inhibitor, underscoring the potential of IL-24 as an anti-cancer agent explored in this study.

This study shows, for the first time, that the molecular mechanism of the cancer-specific killing effect observed in IL-24-treated cells involves a GSK3β-dependent pathway. Also, our study shows that PKA mediates IL-24-induced apoptosis in prostate cancer cell lines. We showed for the first time that IL-24 inhibits GSK3β and involves phosphorylation at serine 9 by protein kinase A (PKA) and threonine 390 by p38 MAPK in prostate cancer cells. Furthermore, our results indicate that IL-24 induces apoptosis, glucose regulation, and mitochondria dysfunction by inhibiting GSK3β. The present study defines the pro-apoptotic mechanism by which IL-24 promoted the death of human prostate cancer cells. Unraveling the biochemical basis of IL-24’s cancer-selective activity is a key to unlocking this molecule’s potential to enhance cancer treatments. Several studies indicate that GSK3β inhibition potentiates the antitumor action of other cytotoxic drugs in vitro and in vivo. Therefore, IL-24-mediated inhibition of GSK3β could be exploited for combination therapy with existing therapies.

## 2. Materials and Methods

### 2.1. Cells and Culture Conditions

All the prostate cancer cell lines in this study, LNCaP (lowly metastatic), DU145 (moderately metastatic), and PC3 (highly metastatic), were purchased from the American Type Culture Collection (ATCC, Manassas, VA, USA), maintained per ATCC protocols and utilized within six months of thawing each vial. All cell lines were cultured in a humidified atmosphere at 37 °C with 5% CO_2_, and media were replaced every alternate day. The PKA inhibitor, H-89 (EMD Millipore, Billerica, MA, USA), was dissolved in dimethyl sulfoxide (DMSO; Sigma-Aldrich, St. Louis, MO, USA) and stored at 10 mM. The final concentration was 10 μM, as previously described [17].

### 2.2. Virus Infection

All the viruses in this paper were purchased from VectorBuilder (Chicago, IL, USA). Stable transfected cells were described previously [18]. After attachment within 24 h, cells were infected with the IL-24 expressing replication-defective adenovirus (Ad.IL-24) or the control, a corresponding empty adenovirus vector lacking exogenous genes (Ad.vector). The adenoviruses were custom-made by VectorBuilder, Inc. (Chicago, IL, USA).

### 2.3. Plasmids and Transfections

Cells were seeded in 6-well plates. After reaching 60–70% confluence, media was replaced with Opti-MEM (Thermo Fisher Scientific Inc., Wilmington, DE, USA), and cells were transfected with either plasmids encoding wild-type GSK3β (Addgene, Watertown, MA, USA, #14753), or GSK3β S9A (Addgene, Watertown, MA, USA, #14754) using Lipofectamine 2000 (Thermo Fisher Scientific Inc., Wilmington, DE, USA) according to the manufacturer’s instructions. The same protocol was conducted with mock vector pcDNA3, which was used as an assay control for all experiments. The plasmid pcDNA3-GSK3β (S9A) and pcDNA3-GSK3β were obtained from Addgene (Addgene, Watertown, MA, USA, plasmid numbers 14754 and 14755) and created by Dr. James Woodgett’s laboratory [38]. GSK3β S9A differs from wild-type in that an alanine has been substituted for serine at residue 9, thereby rendering it constitutively active.

### 2.4. Western Blot Analysis

Protein extracts were prepared with RIPA buffer containing a mixture of protease inhibitors as described. Fifty micrograms of protein were briefly applied to a 12% SDS/PAGE and transferred to nitrocellulose membranes. Membranes were incubated with Odyssey blocking buffer (Li-Cor) before incubation with polyclonal or monoclonal antibodies overnight at 4 °C. The membranes were probed with antibodies to phospho-GSK3β (serine 9), phospho-GSK3β (threonine 390), phospho-glycogen synthase (serine 641), phosphor-PKA substrates, cleaved caspase 3, HA-tag, Bax, and β-actin. Goat anti-rabbit IgG (H + L) 800 CW, goat anti-rabbit (680 RD), or goat anti-mouse (H + L) were applied for 45 min at room temperature (1:25,000, LI-COR) before washing with 1× Phosphate Buffered Saline Tween-20 (PBS-T). Visualization and quantification were carried out with the LI-COR Odyssey scanner (LI-COR Bioscience, Lincoln, NE, USA) and Image Studio software version 5.2 (LI-COR Biosciences Lincoln, NE, USA) as previously described [17].

### 2.5. Viability Assays

Cells were plated in 96-well dishes (2 × 10^3^ cells/well) DMEM containing 10% FBS and allowed to attach for 12 h before treatment(s). Cell growth and viable cell numbers were monitored by 3-(4,5dimethylthiazol-2-yl)-2,5-diphenyltetrazolium bromide (MTT) staining, as previously described [25]. The resulting absorbance measured at 595 nm is directly proportional to the number of viable cells.

### 2.6. Annexin V Binding Assays

Cells were trypsinized, washed once with complete medium and PBS resuspended in 0.5 mL of binding buffer containing 2.5 mmol/L CaCl_2_ and stained with allophycocyanin-labeled Annexin V (Becton Dickinson Biosciences, Palo Alto, CA, USA) and propidium iodide (PI) for 15 min at room temperature. Flow cytometry assays were performed as previously described [17].

### 2.7. Mitochondrial Membrane Potential

Mitochondrial membrane potential was monitored using tetramethylrhodamine ethyl ester (TMRE), as previously described [18], and according to the manufacturer’s instructions (Cayman Chemical Company, Ann Arbor, MI, USA). Briefly, cells were seeded in 12-well plates at 5 × 10^4^ cells/well density. After specific treatments, TMRE was added to each well at a final concentration of 200 nM and incubated at 37 °C for 30 min. Following incubation, cells were washed twice with PBS. Fluorescence intensity, which inversely correlates with mitochondrial membrane depolarization, was immediately quantified using a Tecan Spark 10 M Microplate Reader (Tecan, Männedorf, Switzerland) at excitation and emission wavelengths of 550 nm and 575 nm, respectively. Experiments were repeated three times independently, whereby each biological replicate consisted of a technical quadruplicate.

### 2.8. GSK3β Kinase Assay

To measure the GSK3β beta kinase activity, cells were treated as described in the text; cells were lysed from 10 cm dishes using lysis buffer and centrifuged at 14,000 rpm for 15 min at 4 °C to obtain the soluble fraction, and 100 μg of cell lysate was incubated with a specific GSK3β antibody for 4 h at 4 °C with gentle rotation. After the immunoprecipitation, samples were resuspended in kinase assay buffer and were measured with the GSK3β Kinase Enzyme System kit (Promega Corporation, Madison, WI, USA) per the manufacturer’s instructions. All samples were run in triplicate. Then, the luminescence was measured using a Tecan Spark 10 M Microplate Reader (Tecan, Männedorf, Switzerland).

### 2.9. Intracellular Glucose Measurement

According to the manufacturer’s instructions, intracellular glucose levels were determined using a commercial glucose assay kit (Cell Biolabs, Inc., San Diego, CA, USA). DU145 cells were seeded in 6-well plates at 5 × 10^4^ cells/well density. After 24 h, cells were treated as described in the results section, and the medium was replaced with low-glucose DMEM. After 48 h, the cells were harvested and lysed. Cell viability and count were assessed using the Trypan Blue exclusion method. The lysate was then added to a 96-well microtiter plate. The glucose assay was initiated by adding the reaction mixture, followed by a 30 min incubation at 37 °C under light-protected conditions. Absorbance was measured at 560 nm using a microplate reader. Glucose concentrations were determined by interpolation from a standard curve. To account for potential cell density and viability variations across experimental conditions, glucose consumption data were normalized to the viable cell count. Control wells without cells were included to measure background glucose levels and ensure assay accuracy.

### 2.10. Statistical Analysis

Data are presented as mean ± standard error of the mean (SEM), derived from a minimum of three independent biological replicates. All experiments were conducted in triplicate (N = 3). Statistical analyses were performed using GraphPad Prism^®^ version 10 (GraphPad Software, San Diego, CA, USA) and Microsoft Excel. An unpaired two-tailed Student’s *t*-test was applied to compare the two groups. A two-way analysis of variance (ANOVA) followed by Tukey’s post-hoc test was utilized for multiple group comparisons. Statistical significance was defined as *p* < 0.05, with levels of significance indicated as * *p* < 0.05, ** *p* < 0.01, *** *p* < 0.001, and **** *p* < 0.0001. A 95% confidence interval (CI) was calculated for all estimates. Significant differences relative to the control group were marked with asterisks.

## 3. Results

### 3.1. IL-24-Mediated Activation of PKA in Human Prostate Cancer Cells

**Figure 1:**
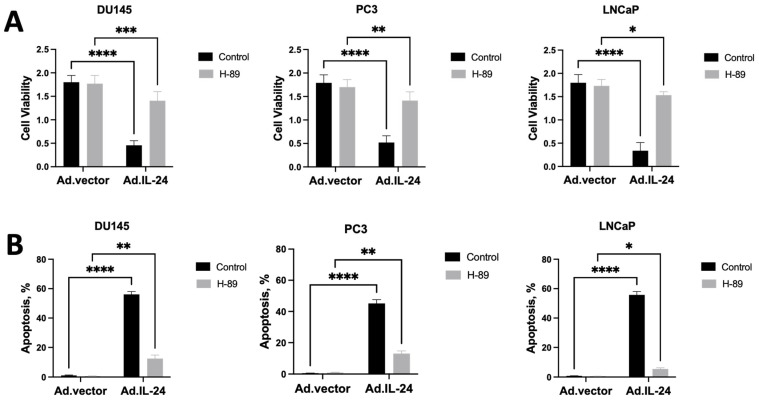

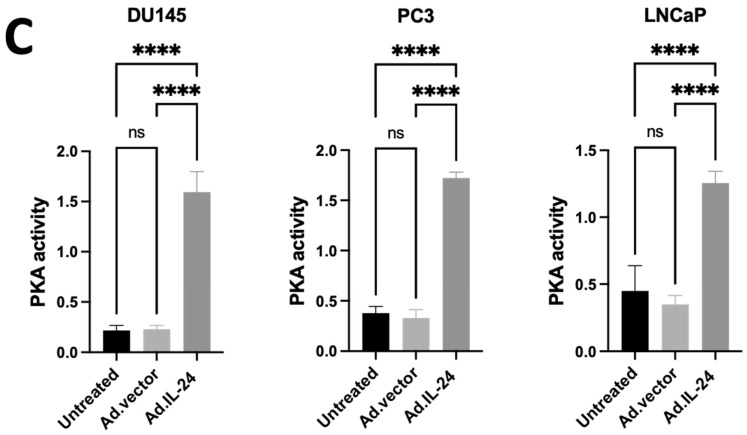
IL-24 induces cell death and apoptosis in prostate cancer cells via PKA. (**A**) Human prostate cancer cells (DU145, PC-3, LNCaP) were treated with Ad.IL-24 or Ad.vector, with or without 10 μM of PKA inhibitor, H-89, and cell viability was determined by the colorimetric proliferation assay five days after treatment. (**B**) Cells were treated as described in A, and the percentage of cells displaying hypodiploidy (Ao), a measure of apoptosis, was determined 48 h later by FACS analysis using the CellQuest Pro software 5.1 (Becton Dickinson, Franklin Lakes, NJ, USA). An average of three independent experiments is shown ± SD; Statistical significance was defined as *p* < 0.05, with levels of significance indicated as * *p* < 0.05, ** *p* < 0.01, *** *p* < 0.001, **** *p* < 0.0001, and ns for not significant. (**C**) Human prostate cancer cells (DU145, PC-3, LNCaP) were treated with Ad.IL-24 or Ad.vector. PKA activity was determined by the colorimetric assay three days after treatment. An average of three independent experiments is shown ± SD.

We have recently shown that IL-24 induces apoptosis by activating the protein kinase A (PKA) pathway in human breast cancer cell lines [17]. First, we determined if IL-24 activates PKA in human prostate cancer cell lines, as shown in human breast cancer cells [17]. We treated several human prostate cancer cell lines (DU145, PC-3, and LNCaP) with IL-24 with or without PKA inhibitor, H-89. Cell viability and induction of apoptosis were measured by 3-(4,5-dimethylthiazol-2-yl)-2,5-diphenyltetrazolium bromide MTT and annexin V-fluorescein isothiocyanate/propidium iodide (FITC/PI) assays, respectively. As shown in Figure 1A,B, H-89 inhibited IL-24-mediated killing in human prostate cancer cell lines. IL-24 activates PKA in all human prostate cancer cell lines (Figure 1C). These results support the hypothesis that IL-24 mediates apoptosis in prostate cancer cells by the PKA signaling pathway.

### 3.2. IL-24-Mediated Inactivation of GSK3β Kinase

**Figure 2:**
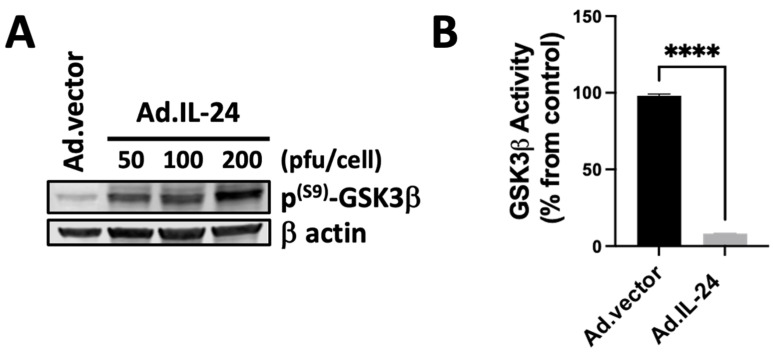
IL-24 inactivates GSK3β kinase in prostate cancer cells in a dosage-dependent manner. (**A**) DU145 cells were treated with Ad.IL-24 (50, 100, or 200 pfu per cell) or Ad.vector (200 pfu per cell). Cells were collected, protein extracts were purified, and western blot analysis was performed to detect phospho-GSK3β (serine 9) and β actin proteins. (**B**) Cells were treated with Ad.IL-24 (200 pfu per cell) or Ad.vector (200 pfu per cell) or control, and the GSK3β activity was determined. An average of three independent experiments is shown ± SD; **** *p* < 0.0001. Numbers represent the ratio of specific treatments to values in control cells.

We hypothesize that GSK3β, a multifunctional kinase involved in cancer progression and cancers resistant to radio-, chemo- and targeted therapy, might play a role in the anti-cancer-specific function of IL-24. Previous studies have shown that PKA can phosphorylate in serine 9 GSK3β and, therefore, inactivate GSK3β [32,34,38]. Furthermore, p38 MAPK, another downstream target of IL-24, can phosphorylate threonine 390 of GSK3β and primes serine 9 phosphorylation of GSK3β by PKA [38,39,40,41,42,43,44]. First, we analyzed whether IL-24 affects the phosphorylation of GSK3β. We treated DU145 human prostate cancer cells with increasing concentrations of IL-24 for 72 h and analyzed the activity and phosphorylation of serine 9 (Figure 2A) of GSK3β. As shown in Figure 2, IL-24 induced phosphorylation of serine 9 of GSK3β in a dosage-dependent manner (Figure 2A). We also measured the activity of GSK3β after IL-24 treatment. As shown in Figure 2B, IL-24 inhibits GSK3β activity. These results show that IL-24 inactivates GSK3β in DU145 human prostate cancer cells. Although our data indicate that PKA inactivates both GSK3α and GSK3β in the DU145 cell line after IL-24 treatment, we decided to focus on GSK3β in this study. This choice was influenced by the literature considering GSK3β a promising target for prostate cancer research and potential therapeutic development [30,31,32]. Nevertheless, we recognize the significance of GSK3α, and future studies should explore its role and the possible impacts of its inhibition in prostate cancer cells.

### 3.3. IL-24-Mediated Activation of Glycogen Synthase and Its Effect on Glucose Levels

**Figure 3:**
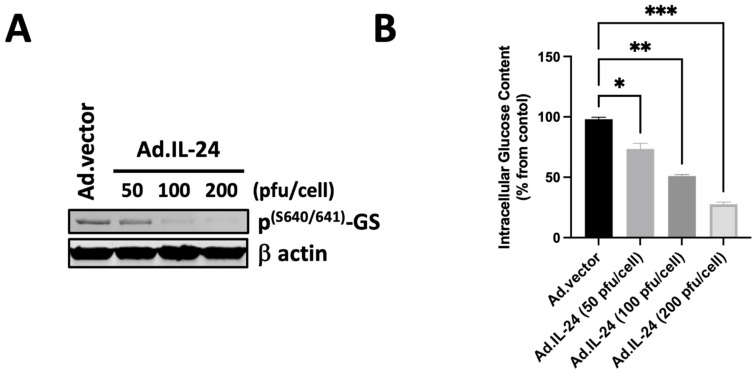
IL-24 activates glycogen synthase and decreases glucose levels. (**A**) DU145 cells were treated with Ad.IL-24 (50, 100, or 200 pfu per cell) or Ad.vector (200 pfu per cell). Cells were collected, protein extracts were purified, and western blot analysis was performed to detect phospho-glycogen synthase (serine 640/641) and β actin proteins. (**B**) Cells were treated as described in A, and the glucose levels were determined. An average of three independent experiments is shown ± SD; Numbers represent the ratio of specific treatments to values in control cells, with significance levels indicated as * *p* < 0.05, ** *p* < 0.01, and *** *p* < 0.001.

It has been established that inhibiting GSK3β impacts glucose metabolism by activating glycogen synthase [33]. The phosphorylation of Ser640/641 (also referred to as site 3a) significantly inactivates glycogen synthase (GS). The slight discrepancy in numbering (Ser640 vs. Ser641) of the phosphorylation site of GS indicates the same functional site in glycogen synthase. GSK3β directly phosphorylates glycogen synthase at Ser640/641. Activating glycogen synthase results in a decrease in glucose levels and an increase in cytoplasmic glycogen storage. Therefore, we first determine whether IL-24 regulates the phosphorylation of Ser640/641 of glycogen synthase. As shown in Figure 3A, IL-24 induces the dephosphorylation of glycogen synthase at Ser640/641 in a dose-dependent manner. We then evaluate the glucose levels following treatment with IL-24. As shown in Figure 3B, IL-24 causes a decrease in glucose levels in a dose-dependent manner. These results support the hypothesis that IL-24 mediates the regulation of glycogen synthase, leading to decreased glucose levels in the DU145 cell line.

### 3.4. IL-24-Mediated Inactivation of GSK3β Kinase in Human Prostate Cancer Cells via PKA

**Figure 4:**
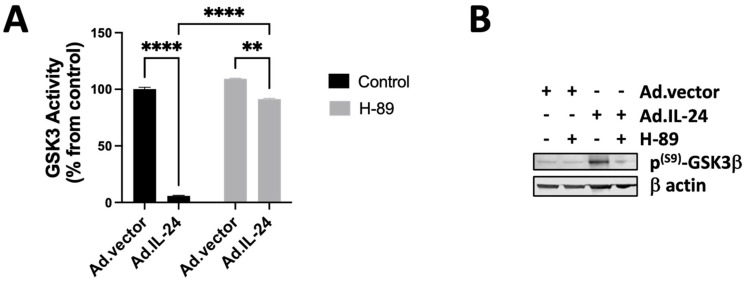
IL-24 inactivates GSK3 and is impaired by a PKA inhibitor, H-89. (**A**) DU145 cells were treated with Ad.vector or Ad.IL-24 and either untreated or treated with 10 μM H-89, and the GSK3β activity was determined. (**B**) Cells were treated as described in A, and western blot analysis was performed with antibodies for phospho-PKA substrates, phospho-GSK3β (serine 9), and β actin. An average of three independent experiments is shown ± SD; Numbers represent the ratio of specific treatments to values in control cells, with levels of significance indicated as ** *p* < 0.01 and **** *p* < 0.0001.

To determine if PKA is involved in the inhibition of GSK3β triggered by IL-24, we analyzed the activity and phosphorylation of serine 9 of GSK3β in DU145 after treatment with IL-24 in the presence or absence of a PKA inhibitor, H-89. DU145 cells were treated with IL-24 with or without the PKA inhibitor. As shown in Figure 4, IL-24 inhibits GSK3β activity via the PKA pathway (Figure 4A) and induces phosphorylation of serine 9 of GSK3β via the PKA pathway (Figure 4B). These findings are consistent with the view that IL-24 inactivates GSK3β through the PKA pathway in human DU145 prostate cancer cells.

### 3.5. IL-24-Dependent Phosphorylation of GSK3β Is Necessary to Mediate Apoptosis in Human Prostate Cancer Cells

**Figure 5:**
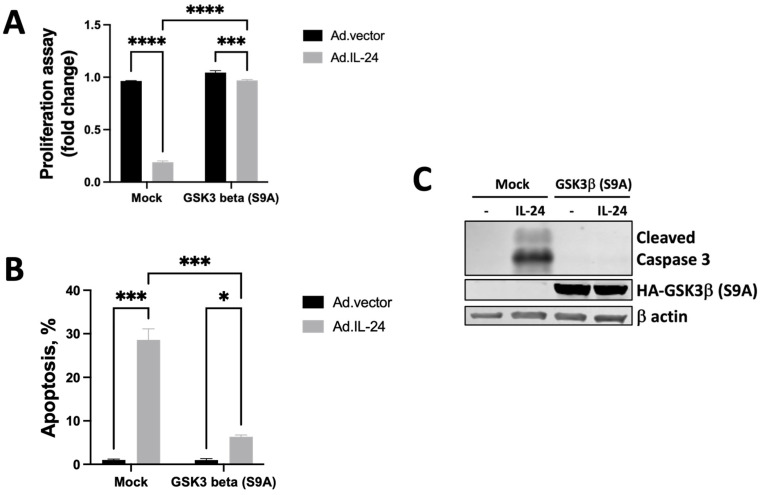
IL-24-dependent phosphorylation of GSK3β is necessary to mediate cell death in DU145 prostate cancer cells. (**A**) DU145 cells were treated with Ad.IL-24, or Ad.vector, in cells that expressed constitutively active GSK3β protein, and cell viability was determined by the colorimetric proliferation assay five days after treatment. An average of three independent experiments is shown ± SD, *** *p* < 0.001 and **** *p* < 0.0001 compared to the control. (**B**) Cells were treated as described in A and assayed for cell death using Annexin V staining to measure apoptosis 48 h later by FACS analysis using the CellQuest software (Becton Dickinson). An average of three independent experiments is shown ± SD, * *p* < 0.05 and *** *p* < 0.001 compared to the control. (**C**) Cells were treated as described in B, and the western blot determined the cleaved caspase 3.

The requirement for GSK3β phosphorylation in serine 9 for IL-24-induced apoptosis was analyzed by IL-24 treatment of human prostate cancer cell lines expressing constitutively active GSK3β [38,39]. The GSK3β S9A mutant variant differs from the wild-type enzyme in that it harbors an alanine substitution for serine at residue 9, resulting in a constitutively active kinase. Compared with the DU145 control cells, constitutively active GSK3β DU145 cells were resistant to the inhibitory action of IL-24 on both cell growth (Figure 5A) and apoptosis (Figure 5B,C). These results show that the phosphorylation in serine 9 of GSK3β is responsible for the inhibitory effect of IL-24 on DU145 cell growth and apoptosis.

### 3.6. IL-24-Dependent Phosphorylation of GSK3β Inhibits Anti-Apoptotic Mechanisms in Mitochondria

**Figure 6:**
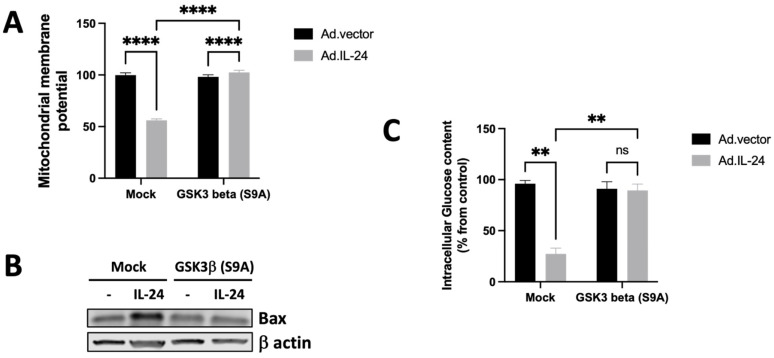
IL-24-dependent phosphorylation of GSK3β is necessary to mediate mitochondrial dysfunction in DU145 prostate cancer cells. (**A**) DU145 cells were treated with Ad.IL-24, or Ad.vector, in cells that expressed constitutively active GSK3β protein, and mitochondrial membrane potential was analyzed by monitoring TMRE fluorescence intensity 72 h after treatment. An average of three independent experiments is shown ± SD, with significance levels indicated as **** *p* < 0.0001. (**B**) Cells were treated as described in (**A**), cells were collected, protein extracts were purified, and subjected to western blot analysis to detect Bax and β actin proteins. (**C**) Cells were treated as described in (**A**), and the glucose levels were determined. An average of three independent experiments is shown ± SD; Numbers represent the ratio of specific treatments to values in control cells; ns: not significant; ** *p* < 0.01.

It has been established that the inhibition of GSK3β impacts glucose metabolism by activating glycogen synthase [33]. The activation of glycogen synthase leads to a drop in glucose levels and an increase in cytoplasmic glycogen storage. A decrease in glucose levels triggers the dissociation of hexokinase activity with mitochondria, resulting in mitochondria-mediated apoptosis. We and others have shown that IL-24 causes mitochondrial-mediated apoptosis [18,22,27,28,29,45]. Therefore, we determined the possible role of GSK3β in the effect of IL-24 on mitochondria dysfunction. DU145 was subjected to IL-24 treatment in the presence or absence of a constitutively active GSK3β, and mitochondrial membrane potential was assessed using the fluorescent dye tetramethylrhodamine ethyl ester (TMRE), which selectively accumulates in mitochondria with intact membrane potential [18]. As shown in Figure 6, IL-24 treatment induced depolarization of mitochondria, as evidenced by decreased TMRE fluorescence intensity, and that effect was blocked in cells that expressed constitutively active GSK3β protein (Figure 6A). We also analyzed the expression of Bax, implicated in the loss of the integrity of the mitochondrial membrane with result in apoptosis, in cells expressing constitutively active GSK3β or control cells. We found that IL-24 treatment increased Bax expression in control cells but not in cells expressing constitutively active GSK3β (Figure 6B). We also tested the glucose levels after treatment with IL-24 in cells expressing constitutively active GSK3β or control cells. We found that IL-24 treatment decreased intracellular glucose levels in control cells but not in cells expressing constitutively active GSK3β (Figure 6C). These results show that the inhibition of GSK3β activity is responsible for the effect of IL-24 on mitochondrial dysfunction, intracellular glucose levels, and Bax expression in the DU145 cell line.

## 4. Discussion

Extensive research within the last decade has shown that IL-24 exhibits tumor suppression activities [2,11,12,13]. These include the inhibition of angiogenesis [3,14], suppression of metastasis and invasion [15,16], and induction of cancer-specific apoptosis [3,17,18,19,20,21,22]. We and others have previously shown that IL-24 exerts its tumor suppression effect in cancer cells by endoplasmic reticulum (ER) stress activation [17,23,24,25], reactive oxygen species (ROS) accumulation [25,26], p38 MAPK activation [17,25,27], mitochondrial dysfunction [18,25,28], and ceramide production while not affecting normal cells [23,29]. Despite extensive research, the precise molecular mechanisms underlying IL-24-induced apoptosis are not fully elucidated. The findings of this study uncover a novel mechanism of IL-24 in apoptosis mediated through cAMP/PKA/GSK3β regulation in prostate cancer cells. Our results indicate that IL-24 inactivates GSK3β through PKA-dependent pathways and activates glycogen synthase. However, further research will be necessary to understand better the precise mechanisms behind these regulations, including co-immunoprecipitation and co-localization experiments, to deepen our knowledge of apoptosis triggered by IL-24.

GSK3β is a well-established negative regulator of β-catenin in Wnt signaling. While β-catenin has been shown to interact with the androgen receptor (AR), the key driver of prostate cancer, extensive studies indicate that GSK3β positively regulates AR activation and promotes tumor progression independently of Wnt signaling. Notably, the selective GSK3β inhibitor GIN reduced clonogenic potential, migration, and the stem-like cell population in various prostate cancer cell lines [46,47,48]. Although GIN inhibition activated the β-catenin/Wnt pathway, its anti-cancer effects remained Wnt-independent. Additionally, GSK3β was shown to be essential for AR-associated gene expression in β-catenin knockdown cells [46]. In vivo studies further demonstrated that GSK3β inhibition decreased metastasis in a mouse xenograft model and suppressed tumor growth [36,48]. Collectively, these findings support GSK3β as a promising therapeutic target in prostate cancer, independent of its role in β-catenin degradation.

The involvement of GSK3β in cancer progression and cancers resistant to radio-, chemo-, and targeted therapy has been recognized [30,31,32,34,35,39,41]. The inactivation of GSK3β has been associated with boosting the effectiveness of cancer treatment drugs, and it also protects tissues from the harmful effects of traditional cancer treatments [39,41]. The work reported here provides direct evidence that inhibiting GSK3β activity with IL-24 induces apoptosis in cancer cells. Specifically, we discovered that IL-24 activates PKA in human prostate cancer cells, induces GSK3β phosphorylation in serine 9 and threonine 390, thereby inactivating its kinase activity, and IL-24-dependent phosphorylation of GSK3β is necessary to mediate apoptosis in human prostate cancer cells through an intrinsic apoptotic pathway. Importantly, we show that the inhibition of GSK3β by IL-24 results in the activation of glycogen synthase, downregulation of intracellular glucose, mitochondria dysfunction, induction of Bax protein, cleaved caspase 3, and induction of apoptosis. Future research is needed to delineate the precise mechanism by which intracellular glucose decreases, especially considering the complex interactions between GSK3 inhibition, glycogen synthase regulation, and PKA activation in cancer cells.

We and others have previously demonstrated that IL-24 induces apoptosis in cancer cells through two primary mechanisms: activating endoplasmic reticulum (ER) stress [17,23,24,25] and causing mitochondrial dysfunction [18,25,28]. The interaction between ER stress and mitochondrial dysfunction creates a feedback loop in which initial ER stress leads to mitochondrial calcium uptake and dysfunction [49,50]. This mitochondrial dysfunction, along with reactive oxygen species (ROS) production, likely worsens ER stress [49,50,51,52]. As a result, this cycle probably rapidly amplifies the apoptotic signal. Notably, the present study indicates that IL-24 reduces intracellular glucose, which may also trigger additional ER stress and mitochondrial dysfunction. The relationship between glucose deprivation, ER stress, and mitochondrial dysfunction in the context of IL-24-induced apoptosis presents a promising area for further investigation. Specifically, quantifying the timing between glucose depletion and the onset of ER stress and mitochondrial dysfunction, as well as exploring the molecular mechanisms linking glucose deprivation to ER stress and mitochondrial dysfunction in this context, would enhance our understanding of IL-24’s apoptotic mechanisms and reveal potential targets for synergistic cancer therapies.

Several studies indicate that GSK3β inhibition potentiates the antitumor action of other cytotoxic drugs in vitro and in vivo, such as Doxorubicin and TRAIL [43,44]. Therefore, IL-24-mediated inhibition of GSK3β could be exploited for combination therapy with existing therapies that rely on the expression of death receptors, such as TRAIL. Subsequent research should aim to precisely delineate the most effective combinations of IL-24 as a GSK3β inhibitor with cytotoxic agents in different cancers, including prostate cancer cells.

## 5. Conclusions

Our findings demonstrate for the first time that IL-24 exerts its pro-apoptotic effects in human prostate cancer cells by activating the PKA pathway and subsequently inhibiting GSK3β. Specifically, IL-24-mediated phosphorylation of GSK3β at serine 9 leads to its inactivation, which influences key downstream processes, including glycogen synthase regulation, alterations in glucose metabolism, mitochondrial dysfunction, and upregulation of Bax. Notably, the necessity of GSK3β inactivation for IL-24-induced apoptosis is confirmed by the resistance of cells expressing constitutively active GSK3β to IL-24-mediated cell death.

Notably, recognizing GSK3β inhibition as a crucial mechanism driving IL-24-mediated cancer-specific apoptosis greatly enhances its therapeutic potential in oncology. Considering the well-documented role of GSK3β in cancer progression and resistance to therapy, our findings indicate that IL-24-based treatments might provide a novel approach to targeting prostate cancer cells while reducing effects on normal tissues. Additional studies are necessary to investigate the potential synergy of IL-24 with current therapeutic strategies and to evaluate its translational potential in clinical contexts.

## Data Availability

Data are contained within the article.
